# A cross-sectional study on the perceived barriers to physical exercise among women in Iraqi Kurdistan Region

**DOI:** 10.1186/s12905-023-02696-3

**Published:** 2023-10-17

**Authors:** Sherzad A. Shabu, Mariwan H. Saka, Dara A. Al-Banna, Sahar M. Zaki, Hamdia M. Ahmed, Nazar P. Shabila

**Affiliations:** 1 Department of Community Medicine, College of Medicine, Hawler Medical University, Erbil, Kurdistan Region Iraq; 2 Department of Medicine, College of Medicine, Hawler Medical University, Erbil, Kurdistan Region Iraq; 3 Department of Nursing, Faculty of Nursing, Tishk International University, Erbil, Iraq; 4 College of Health Sciences, Hawler Medical University, Erbil, Kurdistan Region Iraq; 5 College of Health Sciences, Catholic University in Erbil, Erbil, Kurdistan Region Iraq

**Keywords:** Physical exercise, Women, Barriers, Interpersonal factors, Environment factors

## Abstract

**Background:**

Limited research has investigated the barriers to physical exercise among women in Iraqi Kurdistan Region and other similar Muslim and Middle Eastern societies. This study aimed to determine the prevalence of perceived barriers to physical exercise among women and examine the associations of these barriers with the participants’ sociodemographic characteristics.

**Methods:**

A cross-sectional study was carried out in Erbil, Iraqi Kurdistan Region, from December 2022 to January 2023. A self-administered online survey was designed using Google Forms. A convenience sample of 500 women and girls aged 18–65 years was selected for the study. A questionnaire was designed for data collection, including a list of 21 potential barriers to physical exercise developed based on literature review and experts’ opinions. The barriers were divided into three categories: interpersonal (8 barriers), social environment (8 barriers), and built environment factors (5 barriers). The participants were asked to indicate for each potential barrier whether it was “not really a barrier, somewhat a barrier, or a very important barrier.“ The statistical package for social sciences was used to estimate the prevalence of different barriers and assess their association with sociodemographic characteristics using the Chi-square test.

**Results:**

The prevalence of physical inactivity among the study participants was 68.2%. The most prevalent interpersonal barriers to physical exercise included lack of time (47.4%), followed by fatigue (24%), and cost (22.4%). Regarding social environment factors, work (30.6%), harassment outside (22.2%), not having a friend or family member accompanying (19%), and not being allowed by family (15.4%) were the most prevalent barriers to physical exercise. Lack of footpaths, cycle lanes, or parks (34.4%), limited accessibility of gyms or other exercise facilities (25.8%), and environmental pollution (21%) were the most prevalent built environment factors as barriers to physical exercise.

**Conclusion:**

Women in Iraqi Kurdistan Region experience many barriers to physical exercise. Women require family and social support and awareness about exercise benefits to overcome interpersonal and social environment barriers to physical exercise. Built environment factors are very important barriers and can be reduced by taking appropriate action and adopting necessary policies to provide the required infrastructure and facilities for physical exercise.

## Background

Physical inactivity is extremely prevalent in the Middle East and Arab countries, exceeding 40% in most countries [1, 2]. The prevalence is exceptionally high in Iraq (47%), particularly among women (0.6 male/female ratio in the prevalence of physical inactivity in adults) [1]. Lower physical activity among women in these societies can be attributed to gender norms, including conservative dress unsuitable for physical exercise, the need to be chaperoned in public spaces, and the paucity of women-only fitness facilities [1, 3, 4].

Physical inactivity and sedentary behavior are important modifiable risk factors for metabolic disorders and cardiovascular diseases [5]. There is substantial research evidence on the association of lifelong exercise with a longer health span and preventing chronic diseases or delaying their onsets [6]. A linear relationship exists between physical activity and preventing several chronic diseases and premature mortality. Even relatively minor physical activity can have marked health benefits [7]. Any form of physical activity, including leisure time physical exercise, helps prevent several metabolic disorders, mood disorders, and cancers [8].

The prevalence of physical exercise varies in different populations and different countries. The prevalence is remarkably lower in females than in males. A study from 21 European countries showed that the prevalence of physical exercise in young Europeans is 73.2% for men and 68.3% for women. However, it varied considerably from 60 to 80% across the European countries [9]. In Colombia, 15.7% of women practiced physical exercise irregularly, and only 5.2% regularly practiced physical exercise [10]. In Arab and Middle Eastern countries, people, particularly women, are less physically active. A systematic review from Arab countries showed that the highest prevalence of sufficient physical activity participation among young females was in Kuwait (39.3%) and the lowest in Egypt (4%). In Iraq, the prevalence was 20% [11]. The prevalence of physical exercise has increased considerably in both males and females over the last few decades. For example, the prevalence significantly increased in Estonia from 28.0 to 40.6% in women and 26–44% in men over 18 years (2000 to 2018) [12].

Limited research has examined the level of physical activity and leisure time physical exercise in Iraqi Kurdistan Region, particularly among women. A study from Duhok governorate showed that the prevalence of sufficient leisure time physical exercise in the young population was 16.4% (1.7% in females vs. 31.1% in males). The prevalence of work-related physical activity (55.6%; 31.7% in females vs. 79.2% in males) and transport-related physical activity (48.1%; 19.6% in females vs. 76.5% in males) was much higher, but again it was considerably lower in females [13].

There are many barriers to leisure time physical activity, including the lack of available, accessible, and affordable physical exercise programs that respond to social and cultural needs. Safety, nonsupportive social and cultural norms, and climate concerns are also essential barriers [14]. A study from Singapore showed that the top three barriers to physical exercise were lack of time (65%), fatigue (64%), and pollution (56%). Other barriers included a lack of pavement or parks, cost, and safety concerns [15]. For women, the key barriers are fatigue, absence of child care, health problems, culture, lack of time, and lack of support from family and peers [16, 17]. On the other hand, the most important facilitators of physical exercise in women are weight loss and social and family support [17].

Women in Iraqi Kurdistan Region are better off than their counterparts in the rest of Iraq, especially regarding women’s participation in decision-making and laws against gender discrimination. However, women in the region still face serious challenges, such as patriarchal attitudes toward women’s participation in social, economic, and political life, gender-based violence, and female genital mutilation [18, 19]. Despite good progress in political participation, women in Iraqi Kurdistan Region require more efforts and support to change cultural mindsets to attain equal rights to men. There are still some restrictions on women’s socioeconomic life, especially their mobility in public [20].

Several studies have examined the barriers to physical activity worldwide. However, none have investigated this important health concern in Iraqi Kurdistan Region, particularly among women. Similar studies from other Middle Eastern and Arab countries and similar Muslim and conservative societies are limited. Therefore, the main barriers to physical exercise specific to women in these societies are not appropriately uncovered. Identifying these barriers will help to direct action to increase the engagement of women in physical exercise, which will ultimately improve their health conditions. This study aimed to determine the prevalence of perceived barriers to physical exercise among women and examine the associations of these barriers with the sociodemographic characteristics of the participants.

## Methods

### Design and setting

A cross-sectional study was carried out in Erbil, Iraqi Kurdistan Region, from December 2022 to January 2023. A self-administered online survey was designed using Google Forms.

### Participants

We calculated the sample size using the Epi-info. Based on an estimated prevalence of the main barrier to physical activity, i.e., lack of time, in women in Iraq of 65.3% [15] with a 95% confidence interval and ± 5% precision, a sample size of 348 women was calculated. This sample was increased to 600 to account for non-response.

A convenience sample of women and girls aged 18–65 from Erbil City was invited to participate in the study. The sample was selected with the help of the Center for Research and Education in Women’s Health of Hawler Medical University. The center works with different women’s groups in the community through providing health awareness and education services. Also, the center has networking and is in contact with other women’s groups in the community. The sample was selected from these groups of women.

Women and girls aged 18–65 were included in the study, except for pregnant, breastfeeding, and severely ill women and women with disabilities or mental illness who were excluded.

### Study tool and data collection

A questionnaire was designed for data collection, which included two sections. The first section included information about the participants’ sociodemographic characteristics, such as age, marital status, education level, economic status, and area of residence. The second section included a list of 21 potential barriers to physical exercise. This list was based on the literature review [14, 15, 21] and experts’ opinions. The barriers were divided into three categories: interpersonal (8 barriers), social environment (8 barriers), and built environment factors (5 barriers). The participants were asked to indicate for each potential barrier whether it was “not really a barrier, somewhat a barrier, or a very important barrier.“ The study questionnaire was pilot-tested on 12 participants to assess its clarity, comprehensibility, acceptance, and internal consistency. The reliability was assessed using a test-retest approach. Kappa statistic was calculated, which showed a reliability coefficient of 0.79. Twelve experts in the field evaluated content and face validity. The calculated content validity index was 0.89, and the content validity ratio was 0.90.

The Center for Research and Education in Women’s Health shared the online survey tool with the study sample through email and different women’s social media and WhatsApp groups in the Kurdistan region. The snowballing method was used to recruit a larger sample by asking the contacted women to share the survey tool with other women from family, relatives, and friends. A description of the study and its importance was provided. The participants were requested to provide written online consent after explaining that the participation was voluntary and the anonymity of the study was assured.

### Ethical aspects

Online written informed consent was obtained from the participants before completing the survey questionnaire. The study was conducted in accordance with the Declaration of Helsinki, and the study protocol was approved by the Research Ethics Committee of Hawler Medical University.

### Data processing and statistical analysis

Data were analyzed using the statistical package for the social sciences (version 22). We estimated the prevalence of different barriers to physical exercise. We assessed the association between the different barriers and sociodemographic characteristics using the Chi-square test. A *P* value of *≤* 0.05 was considered statistically significant for all the associations. We addressed missing data by excluding the questionnaires or sections with missing data from the analysis.

**Table 1:** Details of sociodemographic characteristics of the study participants

Characteristic	No.	(%)
**Age group (years)**		
18–25	162	(32.4)
26–35	122	(24.4)
36–45	106	(21.2)
46–55	74	(14.8)
> 55	36	(7.2)
**Ethnic groups**		
Kurd	406	(81.2)
Others (Arab, Turkman, Chaldean)	94	(18.8)
**Marital status**		
Married	259	(51.8)
Single	219	(43.8)
Widow	22	(4.4)
**Number of children (n = 281)**		
0	59	(21.0)
1	44	(15.7)
2	75	(26.7)
3	63	(22.4)
4 and more	40	(14.2)
**Employment**		
Employed	323	(64.6)
Not employed	60	(12.0)
Student	117	(23.4)
**Education level**		
College	343	(68.6)
High school or lower	157	(31.4)
**Area of residence**		
Inside city center	455	(91.0)
Outside city center	45	(9.0)
**Self-rated economic level**		
Low	35	(7.0)
Fair	313	(62.6)
Good	152	(30.4)
**Total**	**500**	**(100.0)**

## Results

A total of 500 participants completed the questionnaire, with a response rate of 83.3%. The mean ± SD age of the participants was 34.4 ± 12.7 years. Of the 500 participants, 32.4% were aged 18–25, 81.2% were Kurds, 51.8% were married, 64.6% were employed, 68.6% had a college of higher education, 91% were city residents, and 62.6% had fair economic level (Table 1).

**Table 2:** Prevalence of perceived barriers to physical exercise in studied women

Barriers	Very important barrier		Somewhat a barrier		Not a barrier	
	No.	(%)	No.	(%)	No.	(%)
**Interpersonal factors**						
I have not been thinking about my ability to exercise	51	(10.2)	122	(24.4)	327	(65.4)
Age	46	(9.2)	111	(22.2)	343	(68.6)
A disease or disability	50	(10.0)	103	(20.6)	347	(69.4)
Cost	112	(22.4)	167	(33.4)	221	(44.2)
Lack of motivation/interest	99	(19.8)	181	(36.2)	220	(44.0)
Young children or family needs	105	(21.0)	171	(34.2)	224	(44.8)
Lack of time	237	(47.4)	187	(37.4)	76	(15.2)
Fatigue	120	(24.0)	235	(47.0)	145	(29.0)
**Social environment factors**						
Lack of self-confidence	62	(12.4)	95	(19.0)	343	(68.6)
Not allowed by family (husband or father/brother)	77	(15.4)	90	(18.0)	333	(66.6)
Not having a friend or family member accompany me in physical exercise	95	(19.0)	188	(37.6)	217	(43.4)
Harassment outside	111	(22.2)	150	(30.0)	239	(47.8)
Work	153	(30.6)	213	(42.6)	134	(26.8)
Worried about my looks when I exercise	46	(9.2)	88	(17.6)	366	(73.2)
Feeling too shy or embarrassed to exercise	70	(14.0)	139	(27.8)	291	(58.2)
Lack of support, e.g., my family or friends do not encourage me to exercise	75	(15.0)	132	(26.4)	293	(58.6)
**Built environment factors**						
Limited accessibility of gym or other exercise facilities (e.g., distance hours, open, availability)	129	(25.8)	191	(38.2)	180	(36.0)
Pollution – e.g., Generators in parks	105	(21.0)	169	(33.8)	226	(45.2)
Lack of footpaths, cycle lanes, or parks	172	(34.4)	165	(33.0)	163	(32.6)
Safety concerns (e.g., street lighting, traffic)	93	(18.6)	168	(33.6)	239	(47.8)
The weather (e.g., wet and hot, cold, rain)	64	(12.8)	199	(39.8)	237	(47.4)

**Table 3:** Association of interpersonal barriers to physical exercise with the sociodemographic characteristics of the studied women

Characteristic	Total no.	Somewhat of a barrier/very important barrier															
		Disease/ disability		Care for kids/family		Lack of time		Cost		Age		Fatigue		Lack of motivation		Inability	
		No.	(%)	No.	(%)	No.	(%)	No.	(%)	No.	(%)	No.	(%)	No.	(%)	No.	(%)
**Age group (years)**																	
18–25	162	46	(28.4)	65	(40.1)	140	(86.4)	99	(61.1)	37	(22.8)	115	(71.0)	99	(61.1)	49	(30.2)
26–35	122	34	(27.9)	73	(59.8)	106	(86.9)	67	(54.9)	31	(25.4)	93	(76.2)	60	(49.2)	42	(34.4)
36–45	106	32	(30.2)	75	(70.8)	92	(86.8)	59	(55.7)	42	(39.6)	78	(73.6)	60	(56.6)	46	(43.4)
46–55	74	28	(37.8)	44	(59.5)	61	(82.4)	39	(52.7)	27	(36.5)	46	(62.2)	46	(62.2)	22	(29.7)
> 55	36	13	(36.1)	19	(52.8)	25	(69.4)	15	(41.7)	20	(55.6)	23	(63.9)	15	(41.7)	14	(38.9)
P-value		0.533		< 0.001		0.092		0.278		0.001		0.226		0.085		0.195	
**Education level**																	
College	343	103	(30.0)	207	(60.3)	298	(86.9)	188	(54.8)	114	(33.2)	237	(69.1)	189	(55.1)	116	(33.8)
High school or lower	157	50	(31.8)	69	(43.9)	126	(80.3)	91	(58.0)	43	(27.4)	118	(75.2)	91	(58.0)	57	(36.3)
P-value		0.682		0.001		0.055		0.510		0.191		0.166		0.550		0.587	
**Marital status**																	
Ever married	281	84	(29.9)	194	(69.0)	240	(85.4)	158	(56.2)	98	(34.9)	196	(69.8)	153	(54.4)	102	(36.3)
Single	219	69	(31.5)	82	(37.4)	184	(84.0)	121	(55.3)	59	(26.9)	159	(72.6)	127	(58.0)	71	(32.4)
P-value		0.698		< 0.001		0.667		0.827		0.058		0.486		0.429		0.366	
**Number of children**																	
0	59	19	(32.2)	26	(44.1)	50	(84.7)	34	(57.6)	13	(22.0)	46	(78.0)	32	(54.2)	20	(33.9)
1	44	14	(31.8)	34	(77.3)	37	(84.1)	28	(63.6)	15	(34.1)	25	(56.8)	26	(59.1)	13	(29.5)
2	75	19	(25.3)	55	(73.3)	62	(82.7)	43	(57.3)	26	(34.7)	49	(65.3)	39	(52.0)	24	(32.0)
3	63	19	(30.2)	51	(81.0)	53	(84.1)	29	(46.0)	26	(41.3)	45	(71.4)	33	(52.4)	28	(44.4)
4 and more	40	13	(32.5)	28	(70.0)	38	(95.0)	24	(60.0)	18	(45.0)	31	(77.5)	23	(57.5)	17	(42.5)
P-value		0.894		< 0.001		0.467		0.412		0.124		0.121		0.935		0.394	
**Employment**																	
Employed	323	102	(31.6)	194	(60.1)	278	(86.1)	172	(53.3)	113	(35.0)	230	(71.2)	177	(54.8)	119	(36.8)
Not employed	60	18	(30.0)	37	(61.7)	43	(71.7)	40	(66.7)	18	30.0)	40	(66.7)	34	(56.7)	21	(35.0)
Student	117	33	(28.2)	45	(38.5)	103	(88.0)	67	(57.3)	26	(22.2)	85	(72.6)	69	(59.0)	33	(28.2)
P-value		0.790		< 0.001		0.009		0.148		0.038		0.702		0.733		0.242	
**Ethnic groups**																	
Kurd	406	107	(26.4)	215	(53.0)	351	(86.5)	230	(56.7)	125	(30.8)	289	(71.2)	219	(53.9)	140	(34.5)
Others	94	46	(48.9)	61	(64.9)	73	(77.7)	49	(52.1)	32	(34.0)	66	(70.2)	61	(64.9)	33	(35.1)
P-value		< 0.001		0.036		0.032		0.426		0.540		0.852		0.054		0.909	
**Area of residence**																	
City center	455	141	(31.0)	252	(55.4)	386	(84.8)	245	(53.8)	149	(32.7)	321	(70.5)	260	(57.1)	159	(34.9)
Outside city	45	12	(26.7)	24	(53.3)	38	(84.4)	34	(75.6)	8	(17.8)	34	(75.6)	20	(44.4)	14	(31.1)
P-value		0.548		0.792		0.944		0.005		0.039		0.480		0.102		0.606	
**Economic status**																	
Fair or low	348	102	(29.3)	187	(53.7)	289	(83.0)	224	(64.4)	111	(31.9)	240	(69.0)	191	(54.9)	114	(32.8)
Good	152	51	(33.6)	89	(58.6)	135	(88.8)	55	(36.2)	46	(30.3)	115	(75.7)	89	(58.6)	59	(38.8)
P-value		0.344		0.319		0.098		< 0.001		0.717		0.129		0.447		0.190	

The prevalence of physical inactivity among the study participants was 68.2%, while the prevalence of engagement in regular leisure-time physical exercise was 14.6%. The most prevalent interpersonal factors as very important barriers to physical exercise included lack of time (47.4%), followed by fatigue (24%), and cost (22.4%). Regarding social environment factors, work (30.6%), harassment outside (22.2%), not having a friend or family member accompanying (19%), and not being allowed by family (15.4%) were the most prevalent very important barriers to physical exercise. Lack of footpaths, cycle lanes, or parks (34.4%), limited accessibility of gym or other exercise facilities (25.8%), and environmental pollution (21%) were the most prevalent built environment factors as very important barriers to physical exercise (Table 2). When taking the average prevalence of all factors in each group, the built environment factors were the most frequently reported barriers (22.5%), followed by interpersonal factors (20.5%) and social environment factors (17.2%).

**Table 4:** Association of social environment barriers to physical exercise with the sociodemographic characteristics of the studied women

Characteristic	Total no.	Somewhat of a barrier/very important barrier															
		Work		Not allowed by family		Harassment outside		Feeling shy		Lack of family support		Lack of self-confidence		Worried about how looks		No friend/family accompanying	
		No.	(%)	No.	(%)	No.	(%)	No.	(%)	No.	(%)	No.	(%)	No.	(%)	No.	(%)
**Age group (years)**																	
18–25	162	108	(66.7)	75	(46.3)	100	(61.7)	80	(49.4)	78	(48.1)	56	(34.6)	44	(27.2)	99	(61.1)
26–35	122	100	(82.0)	40	(32.8)	63	(51.6)	49	(40.2)	52	(42.6)	33	(27.0)	39	(32.0)	65	(53.3)
36–45	106	87	(82.1)	27	(25.5)	48	(45.3)	41	(38.7)	36	(34.0)	32	(30.2)	25	(23.6)	57	(53.8)
46–55	74	53	(71.6)	17	(23.0)	33	(44.6)	30	(40.5)	29	(39.2)	22	(29.7)	16	(21.6)	46	(62.2)
> 55	36	18	(50.0)	8	(22.2)	17	(47.2)	9	(25.0)	12	(33.3)	14	(38.9)	10	(27.8)	16	(44.4)
P-value		< 0.001		< 0.001		0.040		0.072		0.156		0.569		0.515		0.255	
**Education level**																	
College	343	261	(76.1)	107	(31.2)	175	(51.0)	130	(37.9)	141	(41.1)	99	(28.9)	86	(25.1)	196	(57.1)
High school or lower	157	105	(66.9)	60	(38.2)	86	(54.8)	79	(50.3)	66	(42.0)	58	(36.9)	48	(30.6)	87	(55.4)
P-value		0.031		0.122		0.435		0.009		0.845		0.071		0.197		0.717	
**Marital status**																	
Ever married	281	206	(73.3)	81	(28.8)	137	(48.8)	110	(39.1)	111	(39.5)	84	(29.9)	65	(23.1)	157	(55.9)
Single	219	160	(73.1)	86	(39.3)	124	(56.6)	99	(45.2)	96	(43.8)	73	(33.3)	69	(31.5)	126	(57.5)
P-value		0.950		0.014		0.081		0.173		0.329		0.411		0.036		0.710	
**Number of children**																	
0	59	39	(66.1)	16	(27.1)	30	(50.8)	21	(35.6)	24	(40.7)	14	(23.7)	12	(20.3)	32	(54.2)
1	44	31	(70.5)	14	(31.8)	23	(52.3)	18	(40.9)	20	(45.5)	19	(43.2)	7	(15.9)	26	(59.1)
2	75	58	(77.3)	22	(29.3)	33	(44.0)	28	(37.3)	25	(33.3)	20	(26.7)	18	(24.0)	41	(54.7)
3	63	51	(81.0)	18	(28.6)	34	(54.0)	24	(38.1)	26	(41.3)	21	(33.3)	16	(25.4)	37	(58.7)
4 and more	40	27	(67.5)	11	(27.5)	17	(42.5)	19	(47.5)	16	(40.0)	10	(25.0)	12	(30.0)	21	(52.5)
P-value		0.293		0.988		0.679		0.795		0.742		0.202		0.588		0.953	
**Employment**																	
Employed	323	254	(78.6)	94	(29.1)	159	(49.2)	119	(36.8)	123	(38.1)	92	(28.5)	79	(24.5)	176	(54.5)
Not employed	60	27	(45.0)	21	(35.0)	31	(51.7)	29	(48.3)	27	(45.0)	22	(36.7)	15	(25.0)	36	(60.0)
Student	117	85	(72.6)	52	(44.4)	71	(60.7)	61	(52.1)	57	(48.7)	43	(36.8)	40	(34.2)	71	(60.7)
P-value		< 0.001		0.010		0.104		0.009		0.112		0.165		0.119		0.435	
**Ethnic groups**																	
Kurd	406	305	(75.1)	147	(36.2)	214	(52.7)	178	(43.8)	168	(41.4)	125	(30.8)	112	(27.6)	232	(57.1)
Others	94	61	(64.9)	20	(21.3)	47	(50.0)	31	(33.0)	39	(41.5)	32	(34.0)	22	(23.4)	51	(54.3)
P-value		0.044		0.006		0.636		0.054		0.984		0.540		0.409		0.611	
**Area of residence**																	
City center	455	328	(72.1)	153	(33.6)	233	(51.2)	188	(41.3)	188	(41.3)	141	(31.0)	117	(25.7)	256	(56.3)
Outside city	45	38	(84.4)	14	(31.1)	28	(62.2)	21	(46.7)	19	(42.2)	16	(35.6)	17	(37.8)	27	(60.0)
P-value		0.074		0.733		0.158		0.488		0.907		0.529		0.081		0.630	
**Economic status**																	
Fair or low	348	253	(72.7)	128	(36.8)	182	(52.3)	149	(42.8)	156	(44.8)	112	(32.2)	97	(27.9)	204	58.6)
Good	152	113	(74.3)	39	(25.7)	79	(52.0)	60	(39.5)	51	(33.6)	45	(29.6)	37	(24.3)	79	(52.0)
P-value		0.703		0.015		0.947		0.486		0.019		0.568		0.412		0.168	

Table 3 shows the association of interpersonal barriers to physical exercise with the sociodemographic characteristics of the studied women. The disease or disability factor was significantly higher among Kurds than other ethnic groups (P < 0.001). Care for kids and family factors barrier was significantly higher among women aged 36–45 years (P < 0.001), with children (P < 0.001), with higher education level (P = 0.001), being married (P < 0.001), not being a student (P < 0.001), and ethnic groups other than Kurds (P = 0.036). The lack of time factor was significantly higher among employed and students than unemployed (P = 0.009) and among Kurds than in other ethnic groups (P = 0.032). The cost factor was significantly higher among those living outside the city (P = 0.005) and those with fair or low economic levels (P < 0.001). The age factor was significantly higher among the older age participants (P = 0.001), employed (P = 0.038), and those living in the city center (P = 0.039).

**Table 5:** Association of built environment barriers to physical exercise with the sociodemographic characteristics of the studied women

Characteristic	Total no.	Somewhat of a barrier/very important barrier									
		Weather		Pollution		Safety concerns		Limited accessibility		Lack of footpath	
		No.	(%)	No.	(%)	No.	(%)	No.	(%)	No.	(%)
**Age group (years)**											
18–25	162	77	(47.5)	69	(42.6)	69	(42.6)	101	(62.3)	114	(70.4)
26–35	122	72	(59.0)	68	(55.7)	66	(54.1)	78	(63.9)	74	(60.7)
36–45	106	52	(49.1)	68	(64.2)	63	(59.4)	70	(66.0)	73	(68.9)
46–55	74	47	(63.5)	50	(67.6)	44	(59.5)	52	(70.3)	55	(74.3)
> 55	36	15	(41.7)	19	(52.8)	19	(52.8)	19	(52.8)	21	(58.3)
P-value		0.050		0.001		0.041		0.461		0.182	
**Education level**											
College	343	194	(56.6)	206	(60.1)	195	(56.9)	230	(67.1)	236	(68.8)
High school or lower	157	69	(43.9)	68	(43.3)	66	(42.0)	90	(57.3)	101	(64.3)
P-value		0.009		< 0.001		0.002		0.035		0.322	
**Marital status**											
Ever married	281	146	(52.0)	160	(56.9)	153	(54.4)	181	(64.4)	186	(66.2)
Single	219	117	(53.4)	114	(52.1)	108	(49.3)	139	(63.5)	151	(68.9)
P-value		0.744		0.276		0.254		0.828		0.514	
**Number of children**											
0	59	36	(61.0)	32	(54.2)	30	(50.8)	37	(62.7)	44	(74.6)
1	44	20	(45.5)	24	(54.5)	22	(50.0)	27	(61.4)	26	(59.1)
2	75	37	(49.3)	42	(56.0)	43	(57.3)	44	(58.7)	47	(62.7)
3	63	31	(49.2)	34	(54.0)	37	(58.7)	49	(77.8)	44	(69.8)
4 and more	40	22	(55.0)	28	(70.0)	21	(52.5)	24	(60.0)	25	(62.5)
P-value		0.520		0.507		0.838		0.160		0.423	
**Employment**											
Employed	323	182	(56.3)	195	(60.4)	183	(56.7)	214	(66.3)	217	(67.2)
Not employed	60	31	(51.7)	29	(48.3)	30	(50.0)	37	(61.7)	41	(68.3)
Student	117	50	(42.7)	50	(42.7)	48	(41.0)	69	(59.0)	79	(67.5)
P-value		0.041		0.003		0.014		0.344		0.984	
**Ethnic groups**											
Kurd	406	214	(52.7)	217	(53.4)	213	(52.5)	271	(66.7)	275	(67.7)
Others	94	49	(52.1)	57	(60.6)	48	(51.1)	49	(52.1)	62	(66.0)
P-value		0.919		0.207		0.807		0.008		0.741	
**Area of residence**											
City center	455	238	(52.3)	246	(54.1)	239	(52.5)	290	(63.7)	305	(67.0)
Outside city	45	25	(55.6)	28	(62.2)	22	(48.9)	30	(66.7)	32	(71.1)
P-value		0.677		0.294		0.641		0.696		0.578	
**Economic status**											
Fair or low	348	189	(54.3)	188	(54.0)	185	(53.2)	228	(65.5)	243	(69.8)
Good	152	74	(48.7)	86	(56.6)	76	(50.0)	92	(60.5)	94	(61.8)
P-value		0.246		0.597		0.515		0.285		0.080	

Table 4 shows the association of social environment barriers to physical exercise with the sociodemographic characteristics of the studied women. Work factor was significantly higher among the 26–45 years age group (P < 0.001), higher education level (P = 0.031), employed (P < 0.001), and Kurds ethnic group ((P = 0.044). Not allowed by family factor was significantly higher among younger age group (P < 0.001), singles (P = 0.014), non-employed (P = 0.010), Kurds (P = 0.006), and fair-low economic status (P = 0.015). The harassment factor was significantly higher among younger age participants (P = 0.040). Feeling shy was significantly associated with education level (P = 0.009) and employment status (P = 0.009). Lack of support was significantly higher among fair and low economic levels (P = 0.019). Worried about how they look was significantly higher among singles (P = 0.036).

Table 5 shows the association of built environment barriers to physical exercise with the sociodemographic characteristics of the studied women. The weather factor was significantly higher among middle-aged participants (P = 0.050), participants with a college of higher education level (P = 0.009), and employed participants (P = 0.041). Environmental pollution factor was significantly higher among the middle age group (P = 0.001), higher educational level (P < 0.001), and being employed (P = 0.003). Safety concerns factor was significantly higher among the middle age group (P = 0.041), higher education level (P = 0.002), and being employed (P = 0.014). The limited accessibility factor was significantly higher among participants with a college of higher education level (P = 0.035) and Kurds (P = 0.008).

## Discussion

This study showed that the built environment factors were the most frequently reported barriers, followed by interpersonal and social environment factors. The most prevalent interpersonal barriers were lack of time (47.4%), fatigue (24%), and cost (22.4%). The most important social environment barriers included work (30.6%), harassment outside (22.2%), not having a friend or family member accompanying (19%), and not being allowed by family (15.4%). The most prevalent built environment barriers included the lack of footpaths (34.4%), limited accessibility of exercise facilities (25.8%), and environmental pollution (21%).

In the current study, lack of time was the most prevalent interpersonal barrier to physical exercise among women. Other important interpersonal barriers included fatigue and cost. Another study from Singapore also identified time and fatigue as the top barriers to physical activity in the general adult population [15]. Another study from Saudi Arabia also recognized fatigue and lack of time as the main interpersonal barriers among a sample of women with diabetes mellitus [22]. Affordability or cost was also an important barrier in a study on the disadvantaged women population in Canada [14].

Most women, whether single or married, young or old, have many responsibilities, such as taking care of children, cooking, and cleaning at home, besides working or studying. Therefore, they usually feel fatigued and lack time for leisure activities such as physical exercise. Lack of time was significantly higher among employed women and students.

The cost is also an important barrier since participation in physical exercise programs in health/fitness clubs or gyms is relatively expensive in Erbil (minimum of USD100 per month). Women have limited choices, as the culture does not encourage them to practice physical exercise in parks and open spaces. In the current study, the cost factor was particularly important for women from rural areas and those with lower economic status.

In our study, only care for children and family and age interpersonal barriers were significantly associated with the age of the participants. Care for children and the family barrier was more prevalent in women aged 36–45. The age barrier was more prevalent among those 46 and older. A Brazil study showed that older adults and older adults reported intrapersonal barriers to physical exercise more frequently than younger women [23].

This study determined work as the most prevalent social environment barrier to physical exercise, followed by harassment outside, not having a friend or family member accompanying, and not being allowed by family. Nonsupportive cultural and social norms were also important social environment barriers among disadvantaged women in Canada [14]. A systematic review also identified similar social environment barriers to physical exercise among young women, including social support, religious and cultural norms, and safety issues related to sexual harassment and other forms of sexual violence in public spaces [24]. Despite good progress with women’s political and social participation in Kurdistan, many social and cultural factors still restrict women from their rights and freedom. The relatively high prevalence of harassment and not being allowed by family indicates such restriction [19, 20].

Women at a younger age, with lower education, singles, students, non-employed, and lower economic status were more likely not to be allowed by the family to practice physical exercise. Many other social environment barriers were significantly higher among women with lower education levels, employment, and economic status. This finding indicates that women with less empowerment are at higher risk of controlling behavior by their families and husbands. These women might not be at risk of deprivation of physical exercise only. However, they may be vulnerable to other human rights abuses and suffer poor health outcomes [25]. Research has shown that women empowerment programs have contributed to women’s increased motivation to change physical activity behavior [26].

Research has shown a positive relationship between the support for physical exercise from partners and family and engagement in physical exercise [27]. Sometimes partners or families prevent or discourage women from physical exercise for disapproving exercise outfits, thinking that sport is only for men, restricting women from leaving home, fear or worry of neighborhood safety, or even thinking that exercise is harmful for reproduction [28–32].

In this study, the most prevalent built environment barrier was the lack of footpaths, cycle lanes, or parks. This was followed by limited accessibility of gyms or other exercise facilities and environmental pollution. Another study on the adult population in Singapore also identified a lack of footpaths, cycle lanes, or parks, and pollution as the top three barriers to physical activity [15]. Lack of footpaths, cycle lanes, or parks was a significant factor for transport-related physical activity. The study from Canada on disadvantaged women similarly identified lack of accessibility as a key barrier to leisure-time physical activity [14].

Erbil has no special cycle lanes, and the footpaths are generally not in good shape. While walking along roads and streets, people frequently require going on roads and being at risk of road traffic accidents. On the other hand, the number of parks is also limited, and many are not in good condition. Most small parks and gardens near the residential quarters include big generators that provide electricity to the surrounding houses. The noise and pollution from these generators make these gardens and parks inappropriate for physical exercise.

Most built environment barriers were more prevalent among older women, women with higher education, and employed. This might indicate a higher awareness of these groups of women of these barriers. However, employed women and those with limited time and fatigue might find such excuses not to carry out physical exercise.

This study has assessed the main barriers to physical exercise among women in a majority Kurdish and Muslim setting. Other studies have shown high levels of interest but low levels of activity in participating in physical exercise among Muslim women. Muslim women face many barriers and challenges to physical exercise, and progress has been slow. Barriers preventing Muslim women’s participation include religious and cultural barriers, the lack of women-only spaces or facilities, the lack of modest sports attire, and the potential to encounter discrimination [33, 34]. Interventions to encourage the engagement of Muslim women in physical exercise should address the multiple layers of the socioecological model to redress the main barriers at different levels. These different levels of barriers include intrapersonal barriers, such as lack of self-efficacy, motivation, and knowledge; interpersonal barriers, such as lack of social support; and environmental barriers, such as lack of affordable facilities appropriate for the cultural and religious beliefs regarding the participation of women with gender-sensitive and modest dress [35].

This study is the first from Iraq and Iraqi Kurdistan region to investigate the barriers to physical exercise among women. Similar studies from the neighboring countries and other Middle Eastern and Arab countries are also very limited. This study shows the main types and details of the barriers to physical exercise among women in these settings. While recent interest and engagement in physical exercise has increased in the region, women, especially those from conservative and deprived societies, have limited engagement in physical exercise, which could be due to different types of barriers. Determining these barriers can help in taking appropriate actions to overcome these barriers to increase the level of participation of women in physical exercise.

This study has some limitations. The type of the study and sampling method affect the generalizability of the findings. The convenience sampling method is usually used when it is difficult to obtain the list of the population to choose a random sample. The snowball sampling method is more useful when it is difficult to identify respondents and there are stigma issues in the research topic. In the ideal situation, the random sampling method should have been used for this study. However, convenience and snowball sampling methods were used due to the difficulty of obtaining a detailed list of the study population with their contact and directly contacting women in this society based on a random selection. A longitudinal study that applies random sampling and considers confounding variables is needed to provide generalizable findings. This study mixed girls and women for the same barriers. Married and unmarried women have different barriers to physical exercise in the Kurdish and Muslim community contexts. Several valid and reliable questionnaires exist for assessing physical activity barriers, such as the Exercise Benefits/Barriers Scale [36]. However, we decided to use a new tool since the existing tools do not cover all the potential barriers relevant to the Kurdish and Muslim contexts.

## Conclusion

This study shows that women in Iraqi Kurdistan Region experience many barriers to physical exercise. Women need an open and inclusive environment to offer more opportunities for physical exercise. Women require family and social support and awareness about exercise benefits to overcome interpersonal and social environment barriers to physical exercise. Built environment factors are significant barriers and can be addressed by taking appropriate action and adopting necessary policies to provide the required infrastructure and facilities for physical exercise. Culturally responsive available, accessible, and affordable physical exercise opportunities should be provided to women in this region. Future research should be directed towards examining the influence of various barriers and applying strategies to overcome these barriers and enhance physical exercise among women.

## Data Availability

The dataset used for the current study is available in the Mendeley datasets repository (10.17632/2s2pwzzkbx.1).
